# A novel interdomain consortium from a Costa Rican oil well composed of *Methanobacterium cahuitense* sp. nov. and *Desulfomicrobium aggregans* sp. nov.

**DOI:** 10.1007/s00203-023-03533-9

**Published:** 2023-04-13

**Authors:** Linda Dengler, Julia Meier, Andreas Klingl, Laura Nißl, Annett Bellack, Dina Grohmann, Reinhard Rachel, Harald Huber

**Affiliations:** 1 Institute of Microbiology and Archaea Centre, University of Regensburg, Universitätsstrasse 31, 93053 Regensburg, Germany; 2 Plant Development and Electron Microscopy, Biocenter LMU Munich, Planegg-Martinsried, Germany; 3 Centre for Electron Microscopy, Faculty for Biology and Preclinical Medicine, University of Regensburg, Universitätsstrasse 31, 93053 Regensburg, Germany

**Keywords:** Interdomain, Consortium, Biofilm, Methanogens, Sulfate-reducing bacteria

## Abstract

A novel interdomain consortium composed of a methanogenic Archaeon and a sulfate-reducing bacterium was isolated from a microbial biofilm in an oil well in Cahuita National Park, Costa Rica. Both organisms can be grown in pure culture or as stable co-culture. The methanogenic cells were non-motile rods producing CH_4_ exclusively from H_2_/CO_2_. Cells of the sulfate-reducing partner were motile rods forming cell aggregates. They utilized hydrogen, lactate, formate, and pyruvate as electron donors. Electron acceptors were sulfate, thiosulfate, and sulfite. 16S rRNA sequencing revealed 99% gene sequence similarity of strain CaP3V-M-L2A^T^ to *Methanobacterium subterraneum* and 98.5% of strain CaP3V-S-L1A^T^ to *Desulfomicrobium baculatum*. Both strains grew from 20 to 42 °C, pH 5.0–7.5, and 0–4% NaCl. Based on our data, type strains CaP3V-M-L2A^T^ (= DSM 113354^ T^ = JCM 39174^ T^) and CaP3V-S-L1A^T^ (= DSM 113299^ T^ = JCM 39179^ T^) represent novel species which we name *Methanobacterium cahuitense* sp. nov. and *Desulfomicrobium aggregans* sp. nov.

## Introduction

Sulfate-reducing bacteria (SRB) and methanogenic archaea both colonize strictly anoxic biospheres. Herein, the competition for the scarce electron donor H_2_ is often the driving force for the success of one over the other. While some SRB show a certain resistance toward oxygen (Volbeda et al. 2013) and have an extremely high affinity toward the valuable hydrogen, methanogens lack these characteristics (Kristjansson et al. 1982; Kristjansson and Schönheit 1983; Feldewert et al. 2021; Muyzer and Stams 2008). Therefore, methanogens are often outcompeted in biospheres with limited hydrogen yet sulfate-rich conditions. Interestingly, both, methanogens and sulfate reducers, have been found several times in habitats associated with gas storage facilities or petroleum industry, while sulfate-reducing bacteria are often involved in microbiologically influenced corrosion of the corresponding infrastructure (Volbeda et al. 2013; Mori and Harayama 2011; Molíková et al. 2022; Procópio 2022).

Methane-producing archaea represent a morphologically diverse group within the *Euryarchaeota*. Most rod-shaped methanogens are assigned to the genera *Methanobrevibacter*, *Methanothermobacter*, and *Methanobacterium* with *Methanobacterium* as the most diverse group, currently encompassing 24 validly published species. These species were isolated from nearly all over the world, but so far not from Costa Rica. Here, we present the first strain from this area, isolated from a former oil well located very close to the Caribbean Sea.

Most bacterial sulfate reducers cluster within the *Deltaproteobacteria*. The genera *Desulfovibrio* and *Desulfomicrobium* are closely related and were formerly considered as one genus (Rozanova et al. 1988). Herein, *Desulfomicrobium* species are characterized by the presence of the sulfite reductase desulforubidin and the lack of the enzyme desulfoviridin (Rozanova et al. 1988; Lee et al. 1973). Currently, the genus *Desulfomicrobium* includes seven validly published species, of which only one is thermophilic.

Our new *Desulfomicrobium* species (CaP3V-S-L1A^T^) was isolated from the same biofilm as the *Methanobacterium* strain (CaP3V-M-L2A^T^) and both can be grown in pure culture or in a stable co-culture. Therefore, we propose the here described strains CaP3V-M-L2A^T^ and CaP3V-S-L1A^T^ as novel species, *Desulfomicrobium aggregans* sp. nov. and *Methanobacterium cahuitense* sp. nov., respectively.

## Materials and methods

### Sampling and isolation

Both strains were isolated from a natural biofilm, which was extracted from an exploratory oil well in Cahuita National Park, Costa Rica, in September 2016. Sampling technique and location as well as enrichment and cultivation on MS medium were described previously (Dengler et al. 2022).

The initial inoculations were performed using 0.5 mL of environmental sample (containing liquid and natural biofilm particles) in 20 mL medium supplemented with 0.1% acetate (w/v) under H_2_/CO_2_ (80:20 v/v, 300 kPa) gas phase. Initial cell growth of methanogenic cells occurred after two weeks of shaking incubation at 37 °C. Here, free methanogenic cells and floating biofilm particles containing both, methanogenic rods and short, non-fluorescent cells, were found. In order to isolate the methanogens, three subsequent dilution series were carried out. All of them failed, and the culture was still contaminated by SRB. To check, whether growth of one of the organisms is dependent on the other, single-cell isolation of both cell types was performed using an optical tweezer for both strains (Huber et al. 1995).

From here on, the medium for the sulfate-reducing strain CaP3V-S-L1A^T^ was changed to MS-Sulf, which equals MS medium but contains an increased sulfate amount of 0.8 g MgSO_4_ × 7 H_2_O. For the methanogenic strain, a sulfate-free equivalent was used: SMS medium. Here, all sulfate salts were substituted by equimolar amounts of chloride salts (L^−1^): 0.45 g NaCl, 5.00 g NaHCO_3_, 0.083 g MgCl_2_ × 6 H_2_O, 0.225 g KH_2_PO_4_ × 3 H_2_O, 0.3 g K_2_HPO_4_ × 3 H_2_O, 0.18 g NH_4_Cl, 0.06 g CaCl_2_ × 2 H_2_O, 0.0016 g NiCl_2_, 0.0014 g FeCl_2_, 1 mL 0.1% resazurin solution, 1 mL sulfate-free tenfold trace mineral solution, and 1 mL tenfold vitamin solution (Huber and KO. 2006). Again, both media were supplemented with 0.1% acetate (w/v) and inoculated under H_2_/CO_2_ gas atmosphere (80:20 v/v, 300 kPa).

For long-term conservation in our own culture collection, cells were centrifuged under anaerobic conditions (3000 × g, 30 min), re-suspended in their corresponding medium with 5% DMSO. They were then sealed in thin glass capillaries and stored over liquid nitrogen. For short-term storage, logarithmic cell cultures were kept at 4 °C for 2–3 months. Additionally, both strains were deposited within the culture collections of DSMZ and JCM.

For comparative analyses, *Methanobacterium subterraneum* A8p^T^ (DSM 11,074) and *Desulfomicrobium baculatum* X^T^ (DSM 4028) were obtained from the Deutsche Sammlung von Mikroorganismen und Zellkulturen (DSMZ; Braunschweig, Germany). Strains were grown in MS-Sulf medium with H_2_/CO_2_ gas phase (80:20 v/v, 300 kPa) and 17 mM acetate at their optimal growth temperature.

### Phylogenetic analysis

Genomic DNA was isolated using the XS-buffer method (xanthogenate-SDS) (Tillett and Neilan 2000) and 2 mL of exponential cell culture. The 16S rRNA gene was then amplified using the archaeal forward primer 8aF (Eder et al. 1999) and the bacterial forward primer 9bF (Burggraf et al. 1992) together with the universal prokaryotic reverse primer 1512uR (Lane 1991). For the amplification of the *mcrA* gene, the primer pair MRbac1 (Mori and Harayama 2011) and ME2 (Hales et al. 1996) was used. Then, PCR products were purified using the Wizard® Genomic DNA Purification Kit (Promega GmbH, Walldorf) according to the manufacturer’s instructions. After the clean-up, the PCR product was Sanger-sequenced (LGC Genomics GmbH, Berlin). Gene sequences were surveyed using 4Peaks 1.8 (Griekspoor and Groothuis 2005) and aligned with reference sequences in MEGAX 10.1.8 (Tamura et al. 2013; Kumar et al. 2018) using the ClustalW alignment (Thompson et al. 1994). An approximately maximum-likelihood tree was constructed with FastTree 2 (Price et al. 2010) and visualized using iTol (Letunic and Bork 2007).

The G + C content of the total DNA was defined by genome sequencing. Here, library preparation was carried out compliant with Oxford Nanopore Technologies (ONT, Oxford, the United Kingdom) guidelines for native barcoding of genomic DNA (with EXP-NBD104 and SQK-LSK108). Sequencing was conducted on a MinION MK1C (MinKNOW v.20.10.6). Basecalling and demultiplexing were performed using guppy (fast option, qscore cutoff 7, v. 4.2.3), and reads were assembled with flye (v. 2.8.2) (Kolmogorov et al. 2019). The G + C contents were then determined from the contig sequences in R using the Biostrings package (Pagès et al. 2022).

### Morphological and physiological characterization

Gram staining, fluorescence, and phase contrast microscopy were performed as described previously (Dengler et al. 2022). Motility was surveyed for the methanogenic strain CaP3V-M-L2A^T^ at 30–50 °C in two-degree steps under anaerobic conditions using a temperature gradient-forming device (Mora et al. 2014) with a phase contrast microscope (Olympus BX53).

For transmission electron microscopy, exponentially grown cells were chemically fixed with 1% glutaraldehyde (final concentration; v/v) for 10 min at 22 °C and concentrated by centrifugation (4,000 × g, 15 min). 10 µl of cell suspension was placed on copper grids (400-mesh; Plano, Wetzlar, Germany) coated in-house with a 10 nm carbon film, and the samples were subsequently shadowed with Pt/C (15° angle; CFE 50; Cressington). Freeze-etching was performed as described previously (Rachel et al. 2002). Transmission electron micrographs were imaged using a CM12 transmission electron microscope (FEI) operated at 120 keV and fitted with a slow-scan CCD camera (TEM 0124; TVIPS).

For scanning electron microscopy, cells were chemically fixed in cacodylate buffer (50 mM cacodylate, 2 mM MgCl_2_, and pH 7.0) containing 2.5% (v/v) glutaraldehyde. Then, one drop of the fixed culture was loaded on a microscope glass slide, covered with a large cover slip, and immediately frozen in liquid nitrogen. The coverslip was then broken off and the sample was again treated with glutaraldehyde containing cacodylate buffer. After 15, 30, and 60 min of incubation, the supernatant was removed and replaced with fresh buffer. Then, the samples were contrasted with 1% OsO_4_ in cacodylate buffer for one hour and dehydrated in a graded series with 10, 20, 40, 60, 80, and 100% acetone for 10 min each. Complete desiccation was achieved by another 20 and 40 min of incubation with 100% acetone. Afterward, critical point drying was executed with liquid carbon dioxide in a critical point dryer (Polaron CAL 9900). Finally, samples were contrasted by sputter-coating with platinum for 40 s (Baltec SCD 050 supercool sputter coater and imaged with a ZEISS Auriga Crossbeam station (ZEISS, Oberkochen, Germany) in the SEM mode via SE-detection at 2 kV acceleration voltage.

Physiological analyses concerning the optimal NaCl concentration, temperature, pH, and substrate specificity were analyzed in triplicates. Sodium chloride was added in concentrations of 0–5% (w/v) and tested in steps of 0.2% between 0 and 1% and in intervals of 0.5% ascending from 1%. In order to determine the substrates used for methanogenesis by strain CaP3V-M-L2A^T^, the following compounds were tested: acetate (17 mM), formate (22 mM), methanol (31 mM), ethanol (21 mM), 1-propanol (17 mM), 1-butanol (14 mM), 2-propanol (17 mM), 2-butanol (14 mM), methylamine (32 mM), and trimethylamine (17 mM). For the sulfate-reducing strain CaP3V-S-L1A^T^ hydrogen (H_2_/CO_2_) lactate (11 mM), formate (22 mM), pyruvate (11 mM), and ethanol (21 mM) were tested as electron donors and sulfate (10 mM), thiosulfate (9 mM), sulfite (1 mM), and elemental sulfur (1% w/v) as electron acceptors. Fermentative growth was tested on fumarate (9 mM), malate (8 mM), lactate (11 mM), pyruvate (11 mM), succinate (9 mM), and propionate (14 mM). Physiological tests were repeated with the addition of 17 mM acetate if growth was not successful after four weeks of incubation to check whether acetate is required for growth. Molarities equal 0.01% (w/v) final concentration in the medium for sulfite and 0.1% (w/v) for all other substrates.

For the determination of optimal growth, cells were counted in triplicates every 24 h for two weeks using a Thoma counting chamber (depth: 0.02 mm). Due to significant biofilm formation, the optimal growth could only be estimated for strain CaP3V-S-L1A^T^.

In order to examine whether the formation of a stable co-culture is unique to both partners or whether one organism of the community can be substituted by another methanogen or SRB, we performed cross-cultivation experiments with the most closely related species of the novel isolates *Methanobacterium subterraneum* A8p^T^ and *Desulfomicrobium baculatum* X^T^.

## Results and discussion

### Phylogenetic analysis

**Fig. 1:**
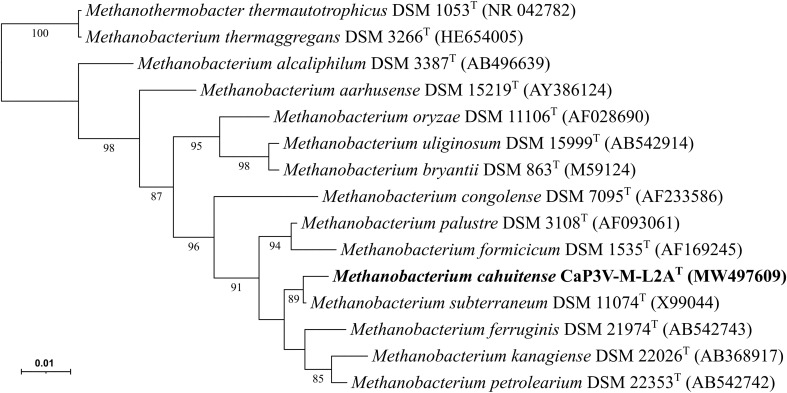
Phylogenetic position of strain CaP3V-M-L2A^T^ based on 16S rRNA gene sequence in relation to other members of the genus *Methanobacterium*. Bootstrap values greater than 85% are displayed. Bar, 1 substitution per 100 nucleotide positions

**Fig. 2:**
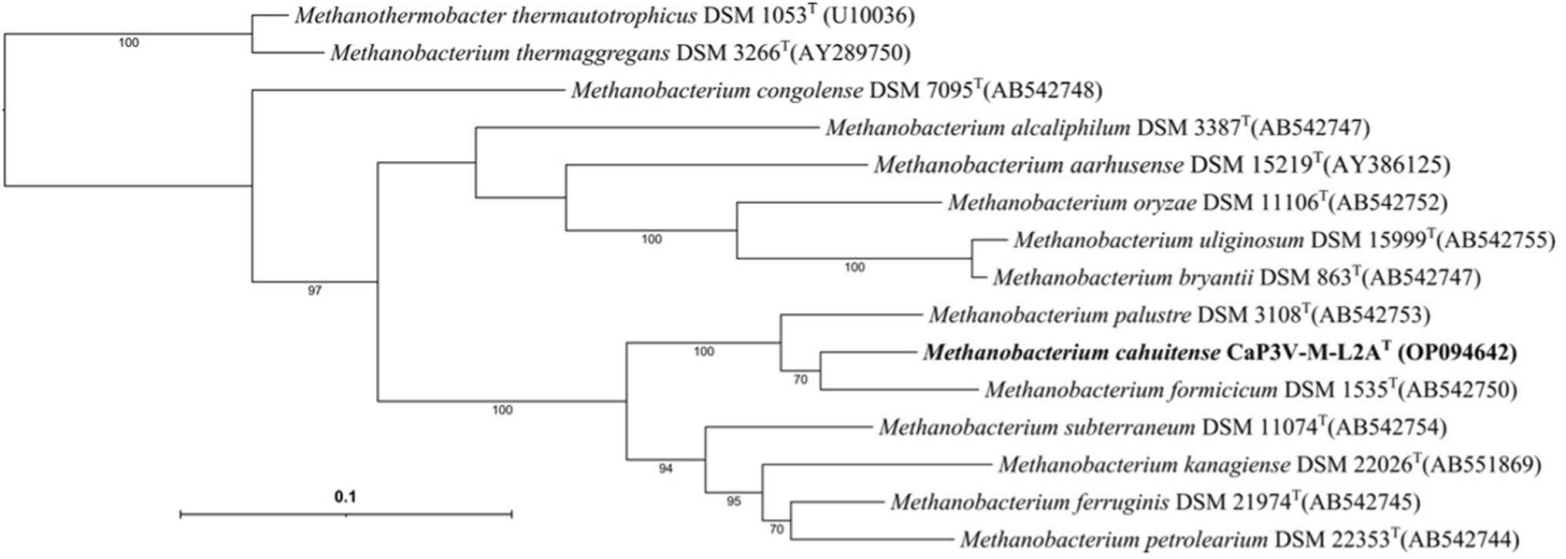
Phylogenetic position of strain CaP3V-M-L2A^T^ based on *mcrA* gene sequence in relation to other members of the genus *Methanobacterium*. Bootstrap values greater than 70% are displayed. Bar, 10 substitutions per 100 nucleotide positions

For the methanogenic strain CaP3V-M-L2A^T^, bidirectional sequencing (LGC Genomics) resulted in a 16S rRNA gene sequence fragment of 1008 b (Fig. 1) and a *mcrA* gene sequence fragment of 1071 bp (Fig. 2). The phylogenetic analysis revealed that the strain belongs to the genus *Methanobacterium*. Its closest relative on 16S rRNA gene sequence level was *Methanobacterium subterraneum* strain A8p with a phylogenetic distance of 0.3%. However, the *mcrA* gene sequence analysis reveals a very clear position between *M. palustre* and *M. formicicum*. The G + C content of the total DNA was 39.3 mol%, which differs significantly from that of *M. subterraneum* Ap8^T^ (G + C content 54.5 mol%) (Kotelnikova et al. 1998).

**Fig. 3:**
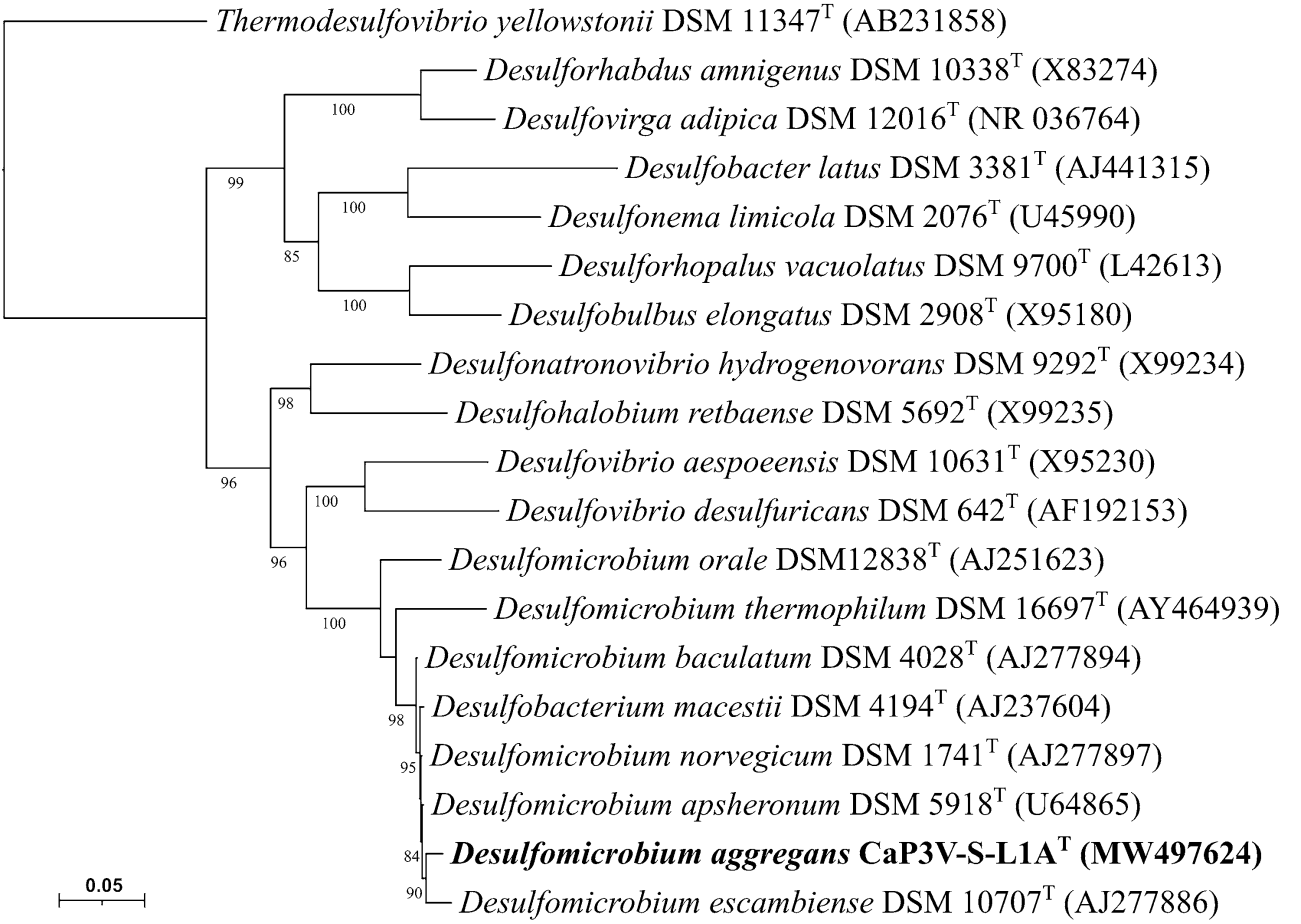
Phylogenetic position of strain CaP3V-S-L1A^T^ based on 16S rRNA gene sequence with all *Desulfomicrobium* species in relation to other members of the *Deltaproteobacteria*. Bootstrap values greater than 80% are displayed. Bar, 5 substitutions per 100 nucleotide positions

The bidirectional sequencing of the sulfate-reducing strain CaP3V-S-L1A^T^ resulted in a 16S rRNA gene sequence fragment of 1415 bp. Here, phylogeny showed that this strain is affiliated with the genus *Desulfomicrobium* (Fig. 3). The most closely related species were *Desulfomicrobium baculatum* H.L21 and *Desulfomicrobium norvegicum* Norway 4 with a phylogenetic distance of 1.48% each. Together with three more species, these closely related species cluster together in the phylogenetic tree. The G + C content of the genomic DNA was 64.5 mol%, which differs significantly from the G + C contents of *D. baculatum* (56.8 mol%) and *D. norvegicum* (56.3 mol%) (Rozanova et al. 1988; Sharak Genthner et al. 1997).

### Morphological and physiological characterization

**Fig. 4:**
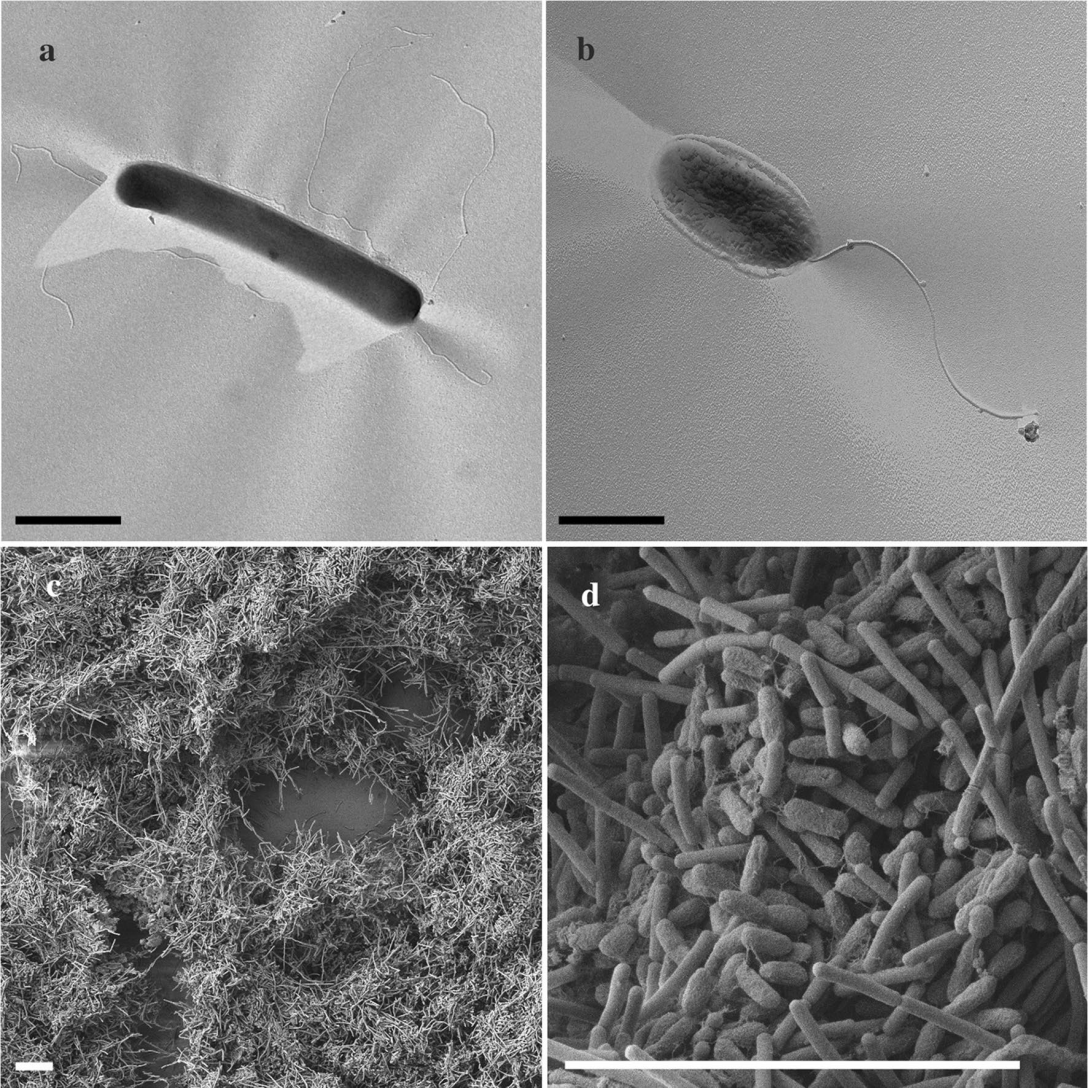
**a**, **b** Transmission electron micrograph of Pt/C shadowed cells of strain **a** CaP3V-M-L2A^T^ and **b** CaP3V-S-L1A^T^ and showing characteristic cell-shapes as well as **a** fimbriae and **b** a flagellum. Bars, 1 µm. **c**, **d** Scanning electron micrograph of the co-culture showing a densely packed cell aggregate. Bars, 10 µm

Cells of strain CaP3V-M-L2A^T^ showed factor F_420_ autofluorescence characteristic for methanogens, stained Gram-positive, and were non-motile. In pure culture, rods occurred as single cells or in chains of 2–6 cells with a diameter of 0.2–0.3 μm and a length of 1.4–20 µm. Electron microscopy revealed a thickened cell wall typical for the pseudomurein components in *Methanobacterium* species and some cells showed cell appendages with a diameter of 5–9 nm (Fig. 4a), reminiscent to fimbriae described for *Methanothermobacter thermautotrophicus* (Thoma et al. 2008).

Strain CaP3V-M-L2A^T^ used H_2_/CO_2_ for methane production, but not acetate, formate, methanol, ethanol, 1-propanol, 1-butanol, 2-propanol, 2-butanol, methylamine, or trimethylamine. Acetate or yeast extract stimulated cell growth on H_2_/CO_2_. This stimulating effect of acetate was already described for *M. formicicum* and *M. bryantii* (Tab. 1). No effect of added acetate was obtained with the other electron donors. Strain CaP3V-M-L2A^T^ had a doubling time of 6 h under optimal conditions.

**Table 1:** Characteristics of strain CaP3V-M-L2A^T^ compared to other validly published species within the genus *Methanobacterium*

	1	2	3	4	5	6	7	8	9	10	11	12
Origin	Oil well	Deep ground water	Sewage sludge	Anaerobic digestor	Marshy soil	Crude oil storage tank	Corroded pipe sediment	Marshy soil	Anaerobic digestor	Lake sediment	Rice field	Marine sediment
Temperature (°C) optimum (range)	37–40 (20–42)	20–40 (3.6–45)	37–45	37–39	33–37 (20–40)	35 (20–40)	40	40 (15–45)	37–42 (25–50)	37	40 (20–42)	45
pH optimum (range)	5.5–7.5 (5–7.5)	7.8–8.8 (6.5–9.2)	ND	6.9–7.2	7.0	6.5 (5.5–9.0)	6.0–8.0 (5.5–9.0)	6.0–8.5	7.2 (5.9–8.2)	8.1–9.1	7.0 (6.0–8.5)	7.5–8.0 (5.0–9.0)
NaCl (%) optimum (range)	0–3 (0–4)	0.2–1.25 (0–1.4)	ND	ND	(0–1.8)	0–4 (0–7)	2 (0–7)	ND	ND	ND	0.5(0–2.3)	(0.3–5.3)
Generation time (h)	6	1.7	ND	19	18	39.5	18.5	11	7.5	7.7	ND	ND
Growth on												
H_2_/CO_2_	+ ^a^	+	+ ^a^	+ ^a^	+	+	+	+	+	+	+	+
Formate	−	+	+	−	+	−	−	−	−	−	−	−
Secondary alcohols	−	−	−	+	+	−	−	−	−	ND	ND	−
Acetate requirement	−	−	−	−	ND	+	−	−	ND	−	ND	−
G + C content (mol%)	39.3 (G)	54.5 (T_m_)	41 (BD)	32.7 (BD)	34.3 (T_m_)	38.3 (LC)	37.6 (LC)	29.4 (T_m_)	39.5 (LC)	57 (BD)	31 (LC)	34.9 (LC)

Strains: 1, CaP3V-M-L2A^T^ (data from the present study); 2, *Methanobacterium subterraneum* A8p^T^ (Kotelnikova et al. 1998); 3*, Methanobacterium formicicum* MF^T^ (Balch et al. 1979); 4,* Methanobacterium bryantii* M.o.H^T^ (Balch et al. 1979); 5, *Methanobacterium palustre* F^T^ (Zellner et al. 1988); 6, *Methanobacterium petrolearium* Mic5c12^T^ (Mori and Harayama 2011); 7, *Methanobacterium ferruginis* Mic6c05^T^ (Mori and Harayama 2011); 8, *Methanobacterium uliginosum* P2St^T^ (König 1984); 9, *Methanobacterium congolense* C^T^ (Cuzin et al. 2001); 10, *Methanobacterium alcaliphilum* WeN4^T^ (Worakit et al. 1986); 11, *Methanobacterium oryzae* FPi^T^ (Joulian et al. 2000); 12, *Methanobacterium aarhusense* H2-LR^T^ (Shlimon et al. 2004)

+ , positive, −negative, *ND* not determined

^a^Acetate stimulates

*G + C content determined using genome data (G), HPLC (LC), buoyant density (BD) or melting point (T_m_)

Cells of strain CaP3V-S-L1A^T^ were Gram-negative, motile rods. Electron microscopy revealed one polar flagellum with a diameter of 17 nm, typical for the genus *Desulfomicrobium* (Fig. 4b). In pure culture, the cells rarely occurred as single cells with a diameter of 0.3–0.5 μm and a length of 1–2.5 µm, but mostly formed dense biofilm aggregates with a diameter of up to 1 cm (Fig. 4c, d). This biofilm was formed under all physiological conditions tested so far. Formation of aggregates or biofilm was not observed for the closest relative, *Desulfomicrobium baculatum*.

Strain CaP3V-S-L1A^T^ used H_2_, lactate, formate, and pyruvate as electron donors but not ethanol. Electron acceptors were sulfate, thiosulfate, and sulfite but not elemental sulfur. Acetate was required for growth on H_2_/CO_2_. Fermentative growth occurred on fumarate and malate but not on pyruvate, lactate, succinate, or propionate. Due to the dense biofilm formation, the doubling time of strain CaP3V-S-L1A^T^ could not be determined reliably.

The growth of both strains CaP3V-M-L2A^T^ and CaP3V-S-L1A^T^ was observed at temperatures ranging from 20 °C to 42 °C. The optimal growth temperature was determined to be 37–40 °C. A pH of 5.0–7.5 supported cell growth and the optimal pH was 5.5–7.5, which is the lowest pH optimum of all *Methanobacterium* species compared in Table 1. Both strains grew at sodium chloride concentrations from 0 to 4% (w/v) and the optimum was 0–3%.

In co-culture, both strains grew in MS-Sulf medium or MS medium with addition of 10 mM sulfate, 17 mM acetate, and H_2_/CO_2_ (80:20) as gas phase. Here, methanogenic rods occurred as planktonic cells or were enclosed in the biofilm of the sulfate reducers (Fig. 4c, d). The SRBs were again densely packed within large cell aggregates. Both, hydrogen sulfide and methane were produced under these conditions. Cross-cultivation experiments with *Methanobacterium subterraneum* and *Desulfomicrobium baculatum* indicated that this interdomain consortium is indeed unique. None of our organisms could grow together in co-culture with the corresponding reference strain under the given conditions (i.e., CaP3V-M-L2A^T^ with *Desulfomicrobium baculatum* and CaP3V-S-L1A^T^ with *Methanobacterium subterraneum*)*. D. baculatum* outcompeted both methanogens *M. subterraneum* and the strain CaP3V-M-L2A^T^, while *M. subterraneum* outcompeted the novel sulfate-reducing isolate CaP3V-S-L1A^T^. The co-culture of the novel isolates exists both as an original co-culture received via dilution series and as an artificial co-culture that was later established by newly combining the two isolates.

The former oil well, where both strains were isolated from, displays an open pond with a continuous flow of gas bubbles streaming to the surface. This stream of presumably natural gas might ensure the constant delivery of gaseous nutrients like hydrogen and carbon dioxide. The pond is furthermore heavily influenced by the surrounding rainforest, and large amounts of leaves and organic matter are degraded therein. Photographic material of the pond can be found in the supplements of our previous publication (Dengler et al. 2022). The degradation processes of organic matter in the habitat deliver CO_2_, acetate, and also further substrates for the fermentative metabolism of the sulfate-reducing isolate. The relatively large sodium chloride range that is tolerated by both organisms could additionally hint on a subsurface connection to the closely located Caribbean Sea (approximate distance: 10–15 m). Such a bridge could also introduce additional sulfates. A drain on the pond enables constant leakage of excess water. The ability to attach to surfaces with cell appendages and to form a biofilm might therefore be extremely useful to stay close to the valuable nutrient influx deriving from the spring.

### Taxonomic conclusion

Based on phylogenetics, and morphological and physiological characteristics, the strains CaP3V-M-L2A^T^ and CaP3V-S-L1A^T^ are considered to display novel species within the genera *Methanobacterium* and *Desulfomicrobium*, respectively (Table 1, Table 2).

**Table 2:** Characteristics of strain CaP3V-S-L1A^T^ compared to all validly described species of the genus Desulfomicrobium

	1	2	3	4	5	6	7
Origin	Oil well	Lake sediment	Harbor	Sulfidic spring	Water with oil deposits	Human oral cavity	River sediment
Size (µm)	0.3–0.5 × 1.0–2.5	0.5–0.7 × 0.9–1.9	0.5–1.0 × 3.0–5.0	0.5–1.0 × 3.0–5.0	0.7–0.9 × 1.4–2.9	0.6–0.8 × 1.8–3.0	0.6 × 1.7–2.2
Temperature (°C) optimum (range)	37–40 (20–42)	28–37	25–30	35	25–30	37 (25–39)	28
pH optimum (range)	5.5–7.5 (5.0–7.5)	7.2	ND	7.2	7.2	ND	ND
NaCl (g/L) optimum (range)	0–30 (0–40)	10	23	13	10	ND	5
Electron acceptors							
Sulfate	+	+	+	+	+	+	+
Thiosulfate	+	+	+	+	+	ND	+
Sulfite	+	+	+	+	+	ND	ND
Sulfur	−	−	+	ND	−	ND	ND
Electron donors							
Hydrogen	+ ^a^	+ ^a^	+ ^a^	+ ^a^	+ ^a^	+ ^a^	+ ^a^
Lactate	+	+	+	+	+	+	+
Formate	+	+ ^a^	+ ^a^	+ ^a^	+ ^a^	+ ^a^	+ ^a^
Pyruvate	+	+	+	+	+	+	+
Ethanol	−	−	+	+	+	+	+
Fermentative growth							
Fumarate	+	+	+	+	+	ND	+
Malate	+	+	+	+	+	ND	+
Pyruvate	−	±	±	ND	+	+	+
G + C content (mol%)	64.5	56.8	56.3	58.0	52.5	59.7	63.6^b^

Strains: 1, CaP3V-S-L1A^T^ (data from the present study); 2, *Desulfomicrobium baculatum* X^T^ (Rozanova et al. 1988); 3,* Desulfomicrobium norvegicum* Norway 4^ T^ (Sharak Genthner et al. 1997); 4,* Desulfomicrobium macestii* M-9^ T^ (Hippe et al. 2003); 5, *Desulfomicrboium apsheronum* 1105^ T^ (Rozanova et al. 1988); 6, *Desulfomicrobium orale* NY678^T^ (Langendijk et al. 2001), 7, *Desulfomicrobium escambiense* ESC1^T^ (Sharak Genthner et al. 1997). Comparable data filled using (Genthner and Devereux 2015) and (Rosenberg et al. 2014)

+ positive; −negative; ± , poor, *ND* not determined

^a^Acetate or YE is required

^b^from Dias et al. 2008 (Dias et al. 2008); original G + C content was reported 59.9 mol%

### Description of *Methanobacterium cahuitense* sp. nov.

*Methanobacterium cahuitense *sp. nov. (ca.hui.ten’se. L. suff. *-ense -ensis* suffix pertaining to/originating from; N.L. neut. adj. *cahuitense* originating from Cahuita National Park, Talamanca, Limón, Costa Rica).

Cells are non-motile, rod-shaped, 0.2–0.3 µm in diameter, and 1.4–20 µm in length, occur as single cells or in chains of up two 6 individual cells. Cells stain Gram-positive. Fimbriae are used for adherence. Temperature range for growth is 20–42 °C (optimum, 37–40 °C). Sodium chloride concentration is 0–4% (w/v) (optimum, 0–3%). pH range for growth is 5.0–7.5 (optimum, pH 5.5–7.5). Doubling time is 6 h. H_2_/CO_2_ used for methanogenesis. Addition of 0.1% acetate or yeast extract enhances growth. Propanol and butanol inhibit growth. G + C content of DNA is 39.3 mol%.

The type strain is CaP3V-M-L2A^T^ (= DSM 113354^ T^ = JCM 39174^ T^), isolated from an oil well in the Cahuita National Park, Costa Rica.

### Description of *Desulfomicrobium aggregans* sp. nov.

*Desulfomicrobium aggregans* sp. nov. (ag’gre.gans. L. part. adj. *aggregans* adding to, aggregating, forming cell aggregates).

Cells are short rods, 0.3–0.5 µm in diameter, 1–2.5 µm in length, and motile. They occur as single cells or in pairs and always form dense cell aggregates, one polar flagellum, and Gram-negative. Growth temperature is 20–42 °C (optimum, 37–40 °C). Sodium chloride span for growth is 0–40 g/L (w/v) (optimum, 0–30 g/L). pH range is 5.0–7.5 (optimum, pH 5.5–7.5). Electron donors are H_2_, lactate, formate, and pyruvate, but not ethanol. Electron acceptors are sulfate, thiosulfate, and sulfite, but not elemental sulfur. Acetate is required when H_2_/CO_2_ is electron donor. Fermentative growth occurs on fumarate and malate, but not on pyruvate, lactate, succinate, or propionate. G + C content of DNA is 64.5 mol%.

The type strain is CaP3V-S-L1A^T^ (= DSM 113299^ T^ = JCM 39179^ T^), isolated from an oil well in the Cahuita National Park, Costa Rica.

## Data Availability

The GenBank/EMBL/DDBJ accession numbers for the 16S rRNA gene sequences of strains CaP3V-M-L2A^T^ and CaP3V-S-L1A^T^ are MW497609 and MW497624 respectively. The corresponding number for the *mcrA* gene sequence of strain CaP3V-M-L2A^T^ is OP094642.
